# F4^+^ ETEC infection and oral immunization with F4 fimbriae elicits an IL-17-dominated immune response

**DOI:** 10.1186/s13567-015-0264-2

**Published:** 2015-10-21

**Authors:** Yu Luo, Ut Van Nguyen, Pedro Y. de la Fe Rodriguez, Bert Devriendt, Eric Cox

**Affiliations:** Laboratory of Veterinary Immunology, Department of Virology, Parasitology and Immunology, Faculty of Veterinary Medicine, Ghent University, Ghent, Belgium; Department of Veterinary Medicine and Zootechnics, Universidad Central “Marta Abreu” de Las Villas, Carretera a Camajuani km 5½, 54830 Santa Clara, Villa Clara Cuba

## Abstract

Enterotoxigenic *Escherichia coli* (ETEC) are an important cause of post-weaning diarrhea (PWD) in piglets. Porcine-specific ETEC strains possess different fimbrial subtypes of which F4 fimbriae are the most frequently associated with ETEC-induced diarrhea in piglets. These F4 fimbriae are potent oral immunogens that induce protective F4-specific IgA antibody secreting cells at intestinal tissues. Recently, T-helper 17 (Th17) cells have been implicated in the protection of the host against extracellular pathogens. However, it remains unknown if Th17 effector responses are needed to clear ETEC infections. In the present study, we aimed to elucidate if ETEC elicits a Th17 response in piglets and if F4 fimbriae trigger a similar response. F4^+^ ETEC infection upregulated IL-17A, IL-17F, IL-21 and IL-23p19, but not IL-12 and IFN-γ mRNA expression in the systemic and mucosal immune system. Similarly, oral immunization with F4 fimbriae triggered a Th17 signature evidenced by an upregulated mRNA expression of IL-17F, RORγt, IL-23p19 and IL-21 in the peripheral blood mononuclear cells (PBMCs). Intriguingly, IL-17A mRNA levels were unaltered. To further evaluate this difference between systemic and mucosal immune responses, we assayed the cytokine mRNA profile of F4 fimbriae stimulated PBMCs. F4 fimbriae induced IL-17A, IL-17F, IL-22 and IL-23p19, but downregulated IL-17B mRNA expression. Altogether, these data indicate a Th17 dominated response upon oral immunization with F4 fimbriae and F4^+^ ETEC infection. Our work also highlights that IL-17B and IL-17F participate in the immune response to protect the host against F4^+^ ETEC infection and could aid in the design of future ETEC vaccines.

**Electronic supplementary material:**

The online version of this article (doi:10.1186/s13567-015-0264-2) contains supplementary material, which is available to authorized users.

## Introduction

In neonatal and recently weaned pigs, ETEC-associated diarrhea is a major cause of illness and mortality and leads to great economic losses in the swine production industry worldwide [1, 2]. ETEC express fimbriae, which are long proteinaceous appendages radiating from the surface of the bacterium. These fimbriae mediate adhesion to host intestinal epithelia through an interaction with specific receptors present on the brush borders of the small intestinal enterocytes, enabling bacterial colonization [3]. Porcine-specific ETEC strains possess five different fimbrial subtypes, of which F4 fimbriae are the most frequently associated with ETEC-induced diarrhea in piglets [4–6]. Recent data indicate F4 fimbriae are not merely involved in adherence, but also play a role in the modulation of the immune system [7, 8]. In addition, these F4 fimbriae are potent mucosal immunogens, since they elicit a fast secretion of F4-specific secretory IgA (SIgA) at the intestinal tissues upon oral administration, protecting piglets against a challenge infection [3, 9–11].

SIgA responses can be generated by both T cell-dependent and T cell-independent pathways [12]. Recently, Th17 cells and their production of IL-17A and IL-21 have been implicated in the induction of SIgA directed against gut-dwelling pathogens [13–15]. This ability to trigger SIgA responses explains their critical function in the host defense against extracellular pathogens such as *Candida albicans*, *Citrobacter rodentium,Salmonella typhimurium, Klebsiella pneumonia*, and *Giardia muris* [16–20]. Effective immunity to pathogens requires T lymphocytes to be endowed with appropriate effector properties. In this context, naive CD4^+^ T cells differentiate into different effector cells and tailor their functions to the nature of the microbial threat. Besides the classical Th1 and Th2 cells, at least two other CD4^+^ T effector lineages have been identified and described, including Th17 and regulatory T cells (Tregs) [21, 22]. In humans and mice, Th17 cells can be induced from naive CD4^+^ T cells with IL-6 and/or IL-21 in the combination of TGF-β, and mainly secrete IL-17A, IL-17F and IL-21 [23]. IL-17 (also known as IL‑17A) is the hallmark cytokine of Th17 cells and is the founding member of the IL-17 cytokine family, which consists of six members: IL‑17A, IL‑17B, IL‑17C, IL‑17D, IL‑17E (also known as IL‑25) and IL‑17F [24], [25]. Among the IL-17 family members, IL-17F shares the highest sequence homology with IL-17A [26]. Although both cytokines can bind to the same receptors, regulate inflammatory responses and are involved in mucosal defense, they show a distinct binding affinity for these receptors and as such different roles in triggering immunity [25]. IL-17E on the other hand triggers Th2 immunity and is involved in the clearance of helminths and allergy [27, 28]. Recently, IL-17C produced by goblet cells and enteroendocrine cells has been proposed to mediate the intestinal inflammation in IBD patients [29]. The function of the other IL-17 cytokines in immunity is still poorly understood.

As in humans, porcine Th17 cells arise from naive CD4^+^ lymphocytes via IL-6 in the context of TGF-β and secrete IL-17A and IL-21 [30]. However, whether Th17 cells or IL-17 cytokine family members participate in the immune response against an ETEC infection in pigs or other species is still unclear. The heat labile toxin (LT) from a human ETEC strain was found to enhance IL-17A production by human PBMCs in response to antigen or mitogen stimulation [31]. Similarly, the IL-17A promoting effect of LT was also reported in mice upon *Helicobacterpylori* infection [32]. In pigs, IL-17A mRNA production in the small intestine was upregulated early during F4^+^ ETEC infection [33]. In contrast, serum IL-17A levels were unaltered early during ETEC infection, while in the intestinal tissues a downregulated IL-17A mRNA production 7 days post infection was observed [34]. However, as F4^+^ ETEC infections are usually cleared within 7–8 days, we hypothesized that a potential Th17 response due to ETEC infection should occur earlier. Thus, in the present study, we addressed if an F4^+^ ETEC infection and oral administration with F4 fimbriae could skew the T helper cell differentiation to a Th17 profile by assessing the mRNA expression profile of key transcription factors and cytokines involved in T cell polarization at systemic and intestinal tissues.

## Materials and methods

The methodology of the animal experiment was approved by the Ethical Committee of the Faculty of Veterinary Medicine, Ghent University (EC2014/01).

### F4^+^ ETEC challenge

Six F4 receptor-positive (F4R^+^) piglets (7–8 week-old, Belgian Landrace) were selected based on the MUC4 TaqMan assay as previously described [35]. Upon arrival in the animal care facilities, all animals were treated orally with colistin (150 000 U/kg of body weight/day; Colivet; Prodivet Pharmaceuticals, Eynatten, Belgium) until 3 days before inoculation to prevent potential ETEC infections due to stress caused by transport and handling of the animals. During the whole trial all pigs had access to water and feed ad libitum. To reduce the bacterial gut flora, the piglets were given orally a broad-spectrum antibiotic (2 mL florfenicol (Nuflor; Schering-Plough, Brussels, Belgium) for each pig for two consecutive days. One day after Nuflor administration, piglets were inoculated with the ETEC reference strain GIS26 (O149:K91:F4ac^+^, LT^+^STa^+^STb^+^) or phosphate buffered saline (PBS) on day 0 (D0) and day 1 (D1). In brief, piglets were sedated with Stressnil (40 mg/mL; Janssen-Cilag, Berchem, Belgium) and the gastric pH was neutralized by intragastric administration of 60 mL NaHCO_3_ (1.4% in distilled water) followed by intragastric administration of 10^10^ GIS26 in 10 mL sterile PBS. Faeces were collected at D0 and D1 and at D2, D3 and D4 post infection to determine F4^+^ ETEC shedding as previously described [9]. The severity of diarrhea was scored daily as previously described [36]. On D4, piglets were euthanized using pentobarbital (Kela NV, Belgium) and intestinal tissues were excised. Jejunal segments with and without Peyer’s patches, ileal segments and mesenteric lymph nodes (MLNs) were collected and washed twice with Krebs-Henseleit buffer (0.12 M NaCl, 0.014 M KCl, 1 mM KH_2_PO_4_, 0.025 M NaHCO_3_, pH 7.4) and once with Krebs-Henseleit buffer containing 1% (v/v) formaldehyde. Next, the MLNs and intestinal samples were frozen in liquid nitrogen and stored at −80 °C until RNA extraction.

### Immunohistochemistry

The intestinal tissues were sampled as described above, washed with Krebs-Henseleit buffer, embedded in methocel (Fluka, Bornem, Belgium), snap-frozen in liquid nitrogen and stored at −80 °C until sectioning. Cryosections (14 μm) were cut and mounted on 3-aminopropyl-triethoxysilane (Sigma-Aldrich, Bornem, Belgium)-coated glass slides. After drying for 30 min at room temperature (RT), the slides were fixed in 4% paraformaldehyde for 20 min at 4 °C and then embedded in 0.1% Triton (Triton™ X-100, Sigma-Aldrich) for 10 min at RT. Slides were washed three times with PBS with gentle agitation and then incubated overnight with anti-CD3 mAb (0.5 μg/mL, mouse IgG_1_, clone PPT3) and biotinylated anti-swine IL-17A polyclonal rabbit antibody (2.5 μg/mL, Kingfisher biotech, St. Paul, MN, USA) in PBS at 4 °C in a humidified chamber. Purified Mouse IgG_1_ (0.5 μg/mL, Life Technologies, Carlsbad, CA, USA) and irrelevant rabbit polyclonal IgG (2.5 μg/mL, ab27472, Abcam, Cambridge, UK) were used as negative control. The next day, the sections were washed and incubated with streptavidin-FITC (2.5 μg/mL, Biolegend, London, UK) and Texas Red-X conjugated goat anti-mouse IgG (H + L) secondary antibody (5 μg/mL, Life Technologies) at RT for 1.5 h. Subsequently, the sections were washed in PBS and then the nuclei were counterstained with Hoechst (10 μg/mL, Sigma-Aldrich) for 15 min at RT. Finally, the slides were mounted in glycerol containing 0.223 M 1,4-diazobicyclo-(2,2,2)-octane (Sigma-Aldrich) and imaged on a Leica TCS SP2 confocal microscope (Leica Microsystems GmbH).

### Isolation of peripheral blood mononuclear cells

Blood was taken on heparin from the jugular vein of piglets on D0 and D1 prior to inoculation and on D2, D3 and D4 post infection. PBMCs were isolated by density gradient centrifugation using Lymphoprep (Axis-shield, Oslo, Norway). Erythrocytes were lysed in ammonium chloride solution. The resulting PBMC fraction was washed twice in ice cold PBS + 1 mM EDTA and counted using a hemocytometer. The viability was confirmed by exclusion of the vital dye Trypan blue. Then, the cells were either put in TRIzol Reagent (1 × 10^7^ cells in 1 mL TRIzol Reagent; Life Technologies) for RNA extraction or cultured at a concentration of 5 × 10^6^ cells/mL in leukocyte medium (RPMI-1640 (Gibco), fetal bovine serum (FBS) (10%) (Gibco), sodium pyruvate (1 mM) (Gibco), l-glutamine (2 mM) (Gibco), penicillin (100 IU/mL), streptomycin (100 μg/mL) (Gibco), and non-essential amino acids (1%)) in the absence or the presence of F4 fimbriae (5 μg/mL) for up to 72 h at 37 °C, 5% CO_2_ in a humidified atmosphere. Next, the cells were collected, lysed in 1 mL TRIzol and stored at −80 °C until RNA extraction. Cell supernatants were collected and stored at −80 °C until further processing.

### RNA extraction and cDNA synthesis

Prior to RNA extraction, frozen tissue sample were homogenized in liquid nitrogen with mortar and pestle. RNases were removed by baking mortar and pestle at least 3 h at 200 °C. Briefly, mortar and pestle were chilled in liquid nitrogen followed by grinding of the tissues until a fine powder was formed. This powder (600–1200 mg) was added to 1.0 mL prewarmed (37 °C) TRIzol Reagent and immediately mixed well. Then RNA was extracted following the manufacturer’s instructions. RNA samples were treated with DNase I (Promega, Madison, WI, USA) and purified with the RNeasy Mini Kit (Qiagen Benelux, Venlo, The Netherlands) according to the manufacturer’s guidelines. The RNA concentration and purity were determined by measuring the optical density at OD_260_/OD_280_ and OD_260_/OD_230_ with a NanoDrop 2000/2000c spectrophotometer (NanoDrop Technologies, Wilmingtom, DE, USA). All samples had OD_260_/OD_280_ ratios between 1.9 and 2.0 and OD_260_/OD_230_ ratios between 1.9 and 2.1. Total RNA (1 μg) was reverse transcribed using Superscript™ III Reverse Transcriptase (200 U; Life Technologies) and random primers (7.5 ng/μL; Life Technologies). To check the synthesis of amplifiable cDNA in the reverse transcription, a conventional PCR step was performed using GAPDH and β-actin specific primers (Table 1).

**Table 1:** List of the primers used in the qPCR assay

Gene	Sequence (5′→3′)	Size (bp)	Ta (°C)	Reference
IL-17A	F:ACTCCAAACGCTTCACCTCAC	234	58	NM_001005729.1
	R:AGCCCACTGTCACCATCACTT			
IL-17B-like	F:CTGGCCAAGAGGAAGTGTGAG	92	60	XM_003124086.1
	R:GGGTCGTGGTTGATGCTGTAG			
IL-17F	F:GAGGCAGCAGCTCGGAAAAT	173	60	NC_010449.4
	R:TCCCGGGTGATGTTGTAATCC			
IL-21	F:GGCACAGTGGCCCATAAATC	124	60	[30]
	R:GCAGCAATTCAGGGTCCAAG			
IL-22	F:TTGACCAGTCCAACTTCCAGCAGC	143	60	XM_001926156.1
	R:GCAGCGCTCTCTCATATTGACTCC			
IL-23p19	F:CCAAGAGAAGAGGGAGATGATGA	107	57	NM_001130236.1
	R:TGCAAGCAGGACTGACTGTTGT			
RORγt	F:TTCAGTACGTGGTGGAGTTC	141	60	[30]
	R:TGTGGTTGTCAGCGTTGTAG			
IL-10	F : CCATGCCCAGCTCAGCACTG	295	60	[65]
	R:CCCATCACTCTCTGCCTTCGG			
IL-13	F:GTCATTGCTCTCACCTGCTT	308	58	[66]
	R:TTGGTGTCTCGGATGTGCTT			
GATA-3	F:GCTCTACCACAAAATGAACGGAC	110	58	NM_001044567.1
	R:TCGTTGTGGTTTGACAGTTTGC			
IL-12p40	F:GGTTTCAGACCCGACGAACTCT	112	60	[67]
	R:CATATGGCCACAATGGGAGATG			
IFN-γ	F:GAGCCAAATTGTCTCCTTCTACT	262	60	[68]
	R:CTGACTTCTCTTCCGCTTTCT			
T-bet	F:TCAATCCTACTGCCCACTAC	151	60	[69]
	R:TTAGGAGACTCTGGGTGAAC			
Foxp3	F:TGCCATTCGCCACAACTT	179	60	NM_001128438.1
	R:CCTGTCCATCCTTCTTTCCTT			
AID	F:AGAAGTTTCAAAGCCTGGGAG	92	57	XM_003126511.1
	R:TCAACCTCATACAGGGGCAAA			
β-Actin	F:TCATCACCATCGGCAACG	133	60	[70]
	R:TTCCTGATGTCCACGTCGC			
GAPDH	F:GGGCATGAACCATGAGAAGT	230	60	[65]
	R:AAGCAGGGATGATGTTCTGG			
RPL19	F:AACTCCCGTCAGCAGATCC	147	60	[69]
	R:AGTACCCTTCCGCTTACCG			

*RORγt*: RAR-related orphan receptor gamma t, *AID*: activation-induced (cytidine) deaminase, *RPL*-*19*: 60S ribosomal protein L19

### Real-time qPCR

Primers (Table 1) were designed using Primer 5 to span an exon–exon junction thereby avoiding amplification of genomic DNA. The primers were purchased from Eurogentec (Liege, Belgium). The amplification efficiency of all the reactions ranged from 94 to 103%. The PCR products were sequenced and subjected to agarose gel electrophoresis to verify their specificity. cDNA was diluted 8x in DEPC-treated ddH_2_O and combined with primer pairs and SYBR Green PCR Master Mix (Applied Biosystems, Warrington, UK) according to the manufacturer’s recommendations. Quantitative PCR (qPCR) assays were run on the StepOnePlus real-time PCR system (Applied Biosystems) with the following cycling conditions: 95 °C for 3 min, followed by 40 cycles of denaturation at 95 °C for 15 s, annealing for 30 s and elongation at 72 °C for 30 s. Fluorescence acquisition was measured at 72 °C and melting curve analysis was done at 65–95 °C with continuous fluorescence acquisition. The stability of the GAPDH, β-actin, 60S ribosomal protein L19 (RPL-19) and Cyclophilin A (CyPA) mRNA expression levels was evaluated by geNorm [37]. We finally selected GAPDH, β-actin and RPL-19 as reference genes. All reactions were performed in triplicate and relative gene transcription values were calculated using the 2^−ΔΔCt^ method and normalized against these three selected reference genes [38].

### Purification of F4 fimbriae

F4 fimbriae were purified from the ETEC reference strain GIS 26 or the IMM01 strain (O147:F4ac^+^LT^+^STb^+^, which lacks flagellin expression) as previously described [9]. Briefly, the bacteria were cultured in tryptone soy broth (Difco Laboratories, Biotrading, Bierbeek, Belgium) at 37 °C for 18 h, collected by centrifugation and washed in sterile PBS. Subsequently, F4 fimbriae were isolated by mechanical shearing of the bacterial suspension followed by centrifugation to remove the remaining bacteria. The fimbriae were precipitated with ammonium sulfate (40% saturation), the pellet was dissolved in PBS and dialysed overnight against PBS at 4 °C. Next, the fimbrial proteins were filtrated (0.22 μm) and the endotoxins were removed by using EndoTrap columns (Hyglos GmbH, Regensburg, Germany) following the manufacturer’s guidelines. After endotoxin removal, the fimbrial solution contained almost no endotoxin (0.24 EU/mL) as determined by the Limulus amebocyte lysate test (Lonza, Walkersville, MD, USA). The protein concentration was determined by the bicinchoninic acid reaction (Sigma-Aldrich) with bovine serum albumin as a standard and the purity was assessed by sodium dodecyl sulphate polyacrylamide gel electrophoresis (SDS-PAGE, 12%).

### Oral immunization with purified F4 fimbriae

Eleven F4 receptor-positive (F4R^+^) piglets (4–5-weeks-old) were selected, housed and treated with antibiotics as described above. The experimental group consisted of six F4 fimbriae seronegative pigs (three Hypor-west and three Large White × Belgian Landrace), while the control group contained five F4 fimbriae seropositive pigs (two Hypor × Pietrain and three Yorkshire × Large White × Landrace). Prior to the oral immunization piglets were deprived of feed and water for 3 h. Purified F4 fimbriae from the GIS26 strain (1 mg in 10 mL PBS) or PBS were administered orally for three subsequent days to the piglets of the experimental or the control group, respectively. Blood was taken from the jugular vein on the day prior to the initial immunization and at D4 and D9 post immunization to isolate PBMCs for RNA extraction as described above. Blood samples of D0 and D9 were also used to measure F4-specific serum IgG and IgA antibodies by ELISA to monitor immunization success [9].

### In vitro culture of PBMCs

Blood was taken from 8–12-week-old healthy conventionally reared, F4 seronegative pigs (Belgian Landrace) and PBMCs were isolated and suspended at a concentration of 5 × 10^6^ cells/mL in leukocyte medium as described above. Subsequently, PBMCs were transferred to a 24-well tissue culture plate and stimulated with 5 μg/mL F4 fimbriae in the presence or absence of polymyxin B (Sigma-Aldrich), endotoxin-free F4 fimbriae (5 μg/mL) or medium at 37 °C, 5% CO_2_ in a humidified atmosphere. The cells and supernatants were harvested at 24, 48 and 72 h after stimulation and stored properly as mentioned above. The mRNA expression profile of the PBMCs was analyzed using qPCR as described above.

### Cytokine ELISA

The secretion of IL-17A, IL-10, IFN-γ and IL-22 in cell-free supernatants was measured using commercial ELISA kits according to the manufacturer’s guidelines (IL-17A and IFN-γ, Kingfisher biotech; IL-10, Life Technologies; IL-22, Sigma-Aldrich).

### Statistical analysis

Statistical analysis was performed with the Mann–Whitney U test or Kruskal–Wallis Test for the independent samples and Friedman’s two-way analysis for the related samples in the SPSS 22 software package. The significance level was set at *p* < 0.05.

## Results

### F4^+^ ETEC infection triggers IL-17 signature responses in PBMCs and small intestinal tissues

**Figure 1:**
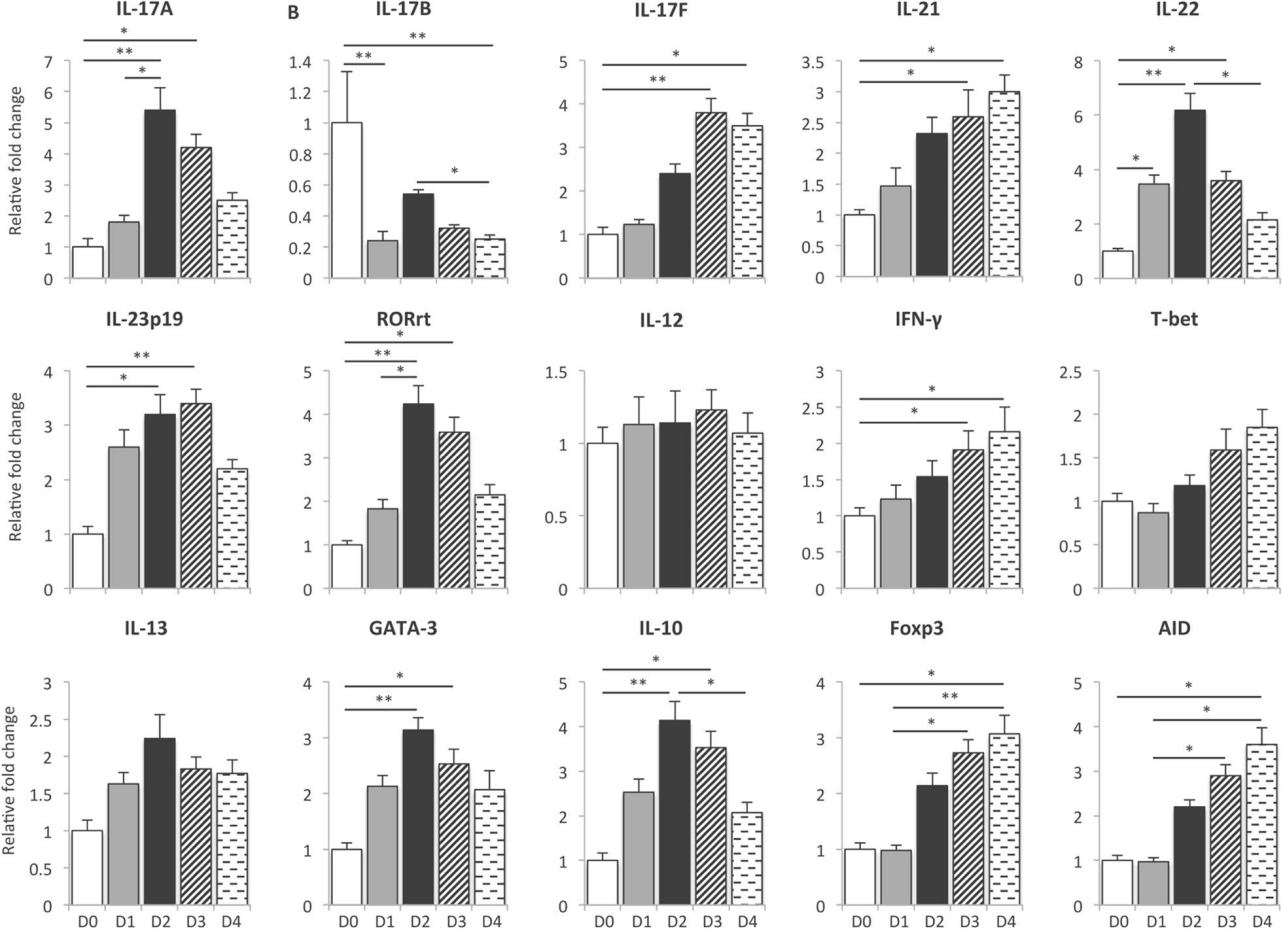
**The mRNA expression profile in PBMCs triggered by F4** ^**+**^ **ETEC infection.** Piglets were infected with F4^+^ ETEC on day 0 (D0) and day 1 (D1). PBMCs were isolated from piglets on D0 until D4 after infection. The mRNA expression in the PBMCs of F4^+^ ETEC infected and control piglets was analyzed by qPCR. The mRNA expression level was normalized to the reference genes and then to the control group for every separate day. Then, the data were plotted relative to D0. Data are presented as the mean ± SEM (*n* = 3 per group). * *p* < 0.05, ** *p* < 0.01.

To analyze the type of immune response elicited by F4^+^ ETEC, we assessed the mRNA expression profile of key cytokines and transcription factors involved in either T cell polarization or their effector functions. Indeed, the mRNA expression levels of Th1 (IFN-γ, IL-12, T-bet), Th2 (IL-13, GATA-3), Th17 (IL-17A, IL-21, IL-22 IL-23p19, orphan nuclear receptor (RORγt)) and regulatory T cells (Foxp3, IL-10) were evaluated in PBMCs and several intestinal tissues. In addition, the mRNA expression of the IL-17 family cytokines IL-17B and IL-17F as well as activation-induced (cytidine) deaminase (AID), a B-cell specific enzyme required for somatic hypermutation and class switch recombination, was assessed. Following F4^+^ ETEC challenge, only one of the three pigs exhibited severe diarrhea, while F4^+^ ETEC shedding was detected in all infected pigs (Additional file 1). Notably, the expression of IL-17A mRNA was significantly increased in PBMCs at D2 and D3 after F4^+^ ETEC infection (Figure 1). In addition, an increased mRNA expression of IL-23p19 and RORγt was also detected these days (Figure 1). Moreover, F4^+^ ETEC infection also increased the mRNA expression of the Th17 cytokines IL-21, IL-22 and IL-17F. In contrast, IL-17B mRNA expression was downregulated in F4^+^ ETEC infected pigs (Figure 1). With regard to the Th1-related genes, only the mRNA expression of IFN-γ was upregulated at D3 and D4, while the mRNA expression of the Th1-related transcription factor T-bet and the Th1-inducing cytokine IL-12 was not influenced by F4^+^ ETEC infection. ETEC infection also significantly enhanced the mRNA expression of the Th2-related transcription factor GATA-3 and the Tregs-related genes Foxp3 and IL-10. Also the AID mRNA levels increased by F4^+^ ETEC in a time-dependent manner in the PBMCs compared to control pigs, which could indicate the presence of pathogen-specific circulating B cells undergoing class switching.

**Figure 2:**
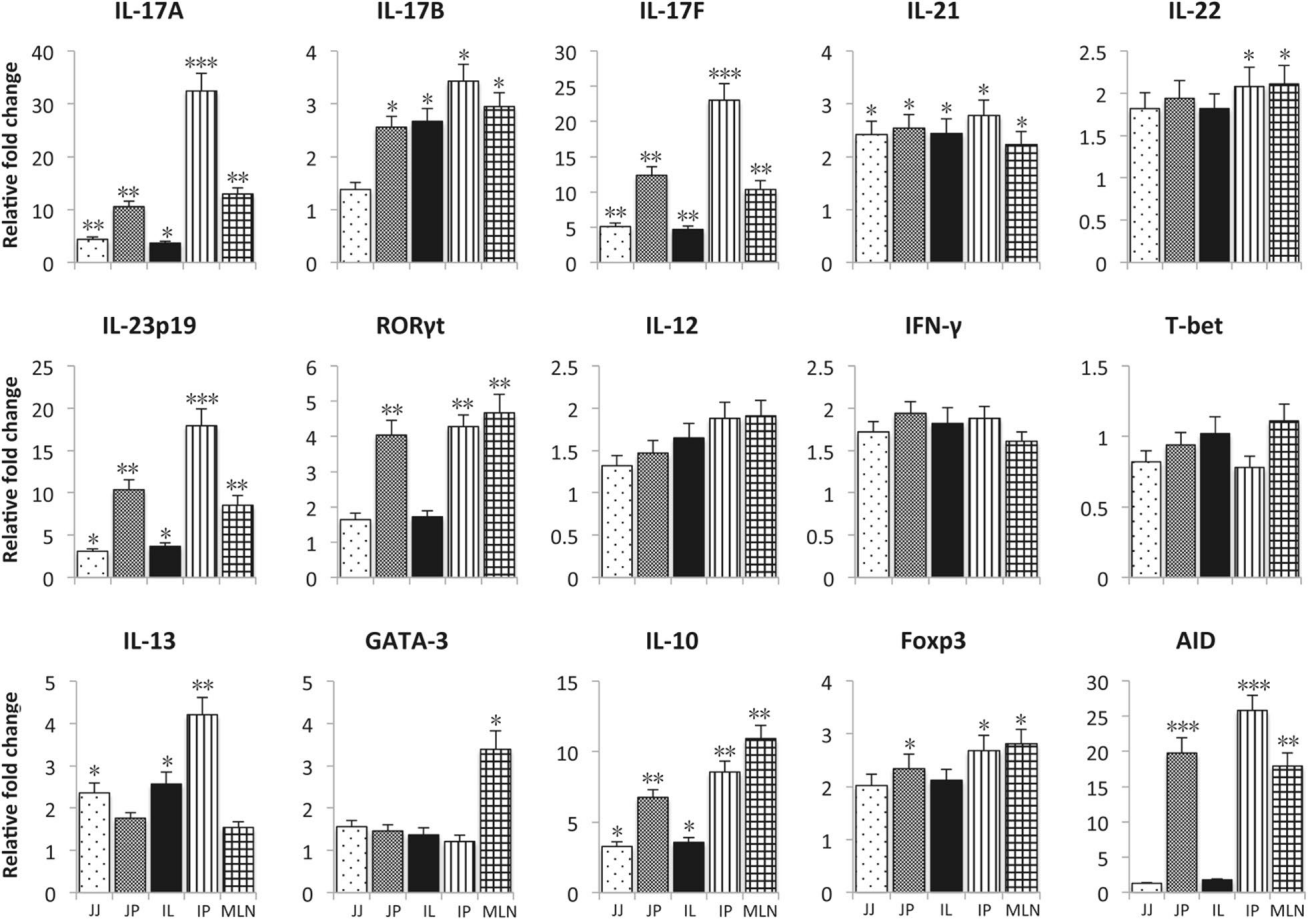
**F4** ^**+**^ **ETEC infection induced mRNA expression profile in intestinal tissues**. The F4^+^ ETEC infection was performed on day 0 (D0) and day 1 (D1). Intestinal samples were collected on D4. The mRNA expression in intestinal tissues of F4^+^ ETEC infected or control pigs was analyzed by qPCR. The mRNA expression was normalized to the reference genes and then to the control group for all separate intestinal tissues. Data are presented as the mean ± SEM (*n* = 3 per group). JJ: jejunum without Peyer’s patches, JP: jejunum with Peyer’s patches, IL: ileum without Peyer’s patches, IP: ileum with Peyer’s patches, MLN: mesenteric lymph nodes. * indicates significant differences as compared to the control group, * *p* < 0.05, ** *p* < 0.01, *** *p* < 0.001.

We further compared the expression profiles of these transcription factors and cytokines in several intestinal tissues on D4 after F4^+^ ETEC infection. As shown in Figure 2, the fold changes of almost all the examined transcripts were higher in the Peyer’s patches (PP) and MLNs than those in the jejunal and ileal lamina propria after F4^+^ ETEC infection. Interestingly, these transcripts also displayed higher expression in the PP and MLN of control pigs (Additional file 2). Similar to PBMCs, F4^+^ ETEC infection significantly increased IL-17A, IL-17F, IL-21 and IL-23p19 mRNA levels in all examined tissues as compared to control pigs (Figure 2). In addition, IL-17A, IL-17F and IL-23p19 transcripts were strongly induced in PP and MLN. In contrast to PBMCs, IL-17B mRNA expression was upregulated upon F4^+^ ETEC infection in all intestinal tissues except jejunum (JJ). No significant difference in IL-22 mRNA expression was observed in jejunal and ileal lamina propria, whereas a small upregulation was found in the ileal PP and MLN in F4^+^ ETEC infected pigs as compared to control pigs. Regarding the Th2-related genes, we observed upregulated IL-13 and GATA-3 mRNA levels with the highest change in the ileal PP and MLN, respectively. Similar to the systemic immune system, F4^+^ ETEC infection did not alter the intestinal mRNA expression of IL-12, IFN-γ and T-bet (Figure 2), while both Foxp3 and IL-10 mRNA levels were significantly upregulated, especially in the PP and MLN. Interestingly, mRNA expression of AID was highly upregulated upon F4^+^ ETEC infection in the PP and MLN, indicating active class switching in those tissues.

### F4^+^ ETEC infection increased CD3^+^IL-17A^+^ T cells in the intestinal tissue

**Figure 3:**
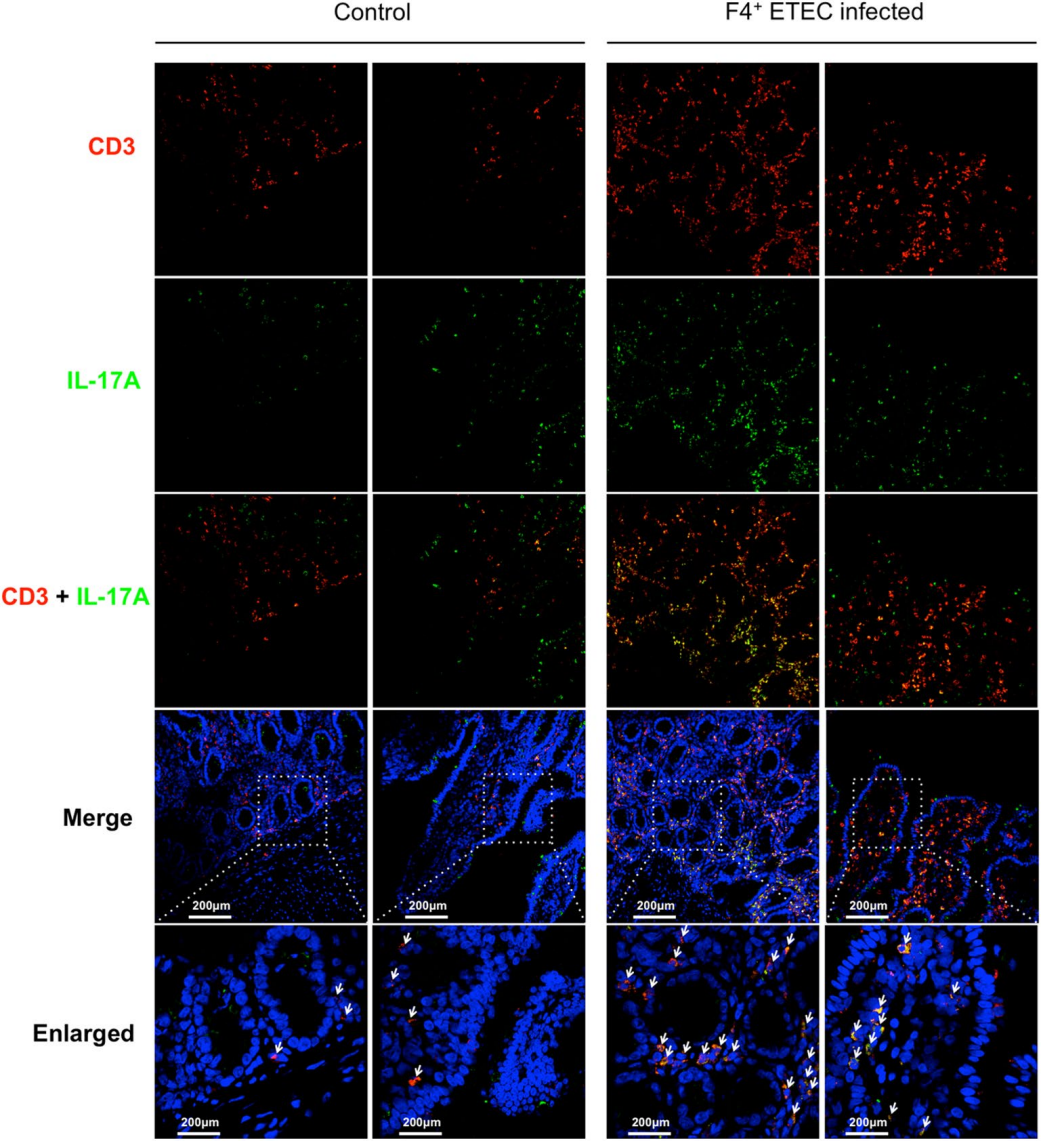
**Increased presence of IL-17A** ^**+**^ **T cells in the ileum of F4** ^**+**^ **ETEC infected piglets.** Cryosections were stained with anti-IL-17A (FITC, green) and anti-CD3 mAbs (Texas Red-X, red). The nuclei were counterstained with Hoechst (blue). Images are representative for all piglets in both groups. The arrows indicate colocalization of CD3 and IL-17A.

Given the high expression of IL-17A mRNA in the ileal PP of F4^+^ ETEC infected piglets, we assessed if this correlated with an influx of IL-17A^+^ T cells in that tissue. Immunofluorescence analysis of ileal tissue clearly showed an increase in CD3^+^IL-17A^+^ cells in the crypts and villi in F4^+^ ETEC infected piglets as compared to controls. In contrast, colocalization of CD3 and IL-17A was rarely observed in the tissue of control pigs (Figure 3).

### Oral immunization of piglets with F4 fimbriae triggered systemic IL-17 responses

**Figure 4:**
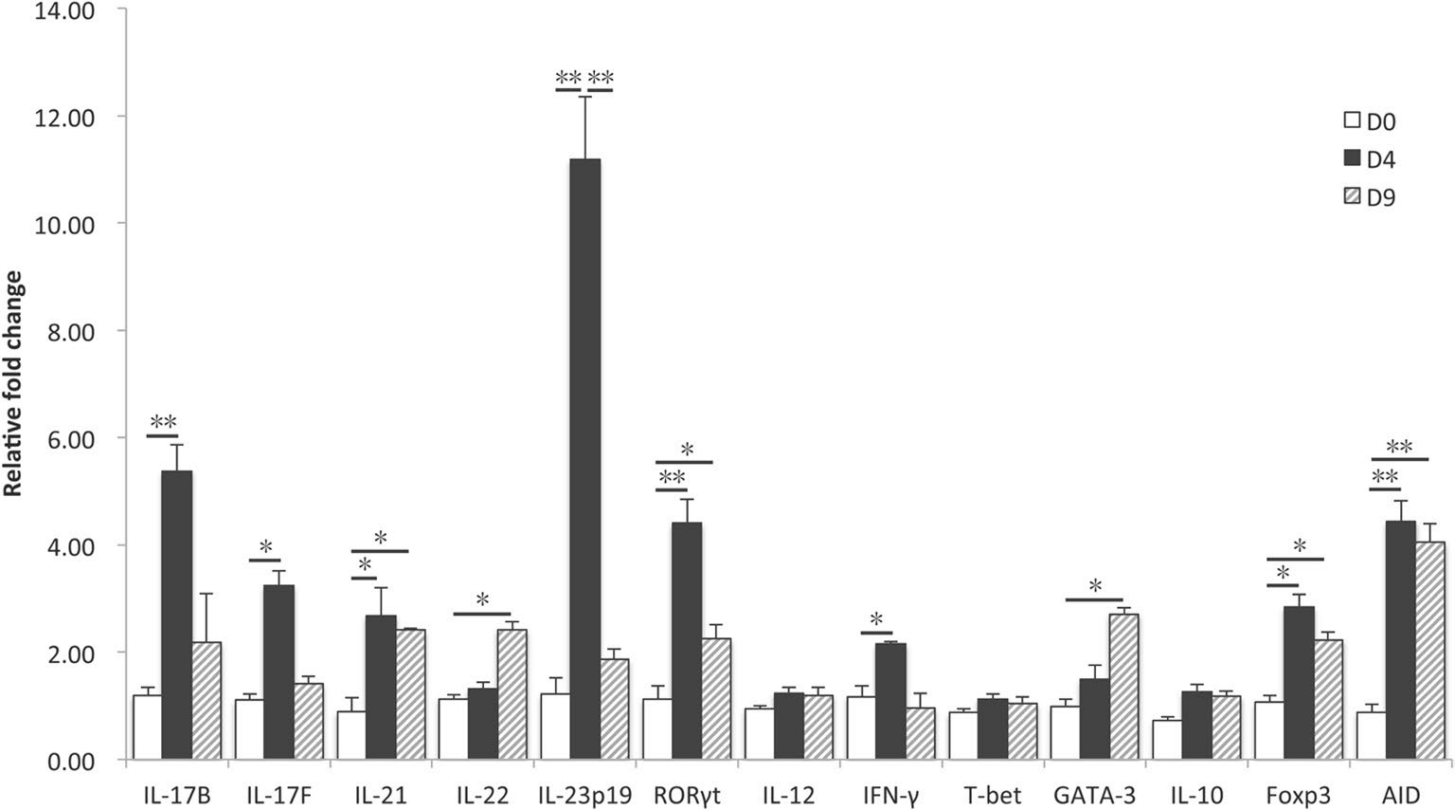
**F4 fimbriae trigger mRNA expression of IL-17 cytokines in PBMCs upon oral immunization.** The piglets were immunized with 1 mg F4 fimbriae on day 0 (D0), D1 and D2. PBMCs were isolated from piglets on D0 before immunization and on D4 and D9 after immunization. The mRNA expression in PBMCs isolated from F4-immunized or control piglets was analyzed by qPCR. The mRNA expression level was normalized to the reference genes and then to the control group for every separate day. Data are presented as the mean ± SEM (*n* = 5 per group). * *p* < 0.05, ** *p* < 0.01.

We detected robust Th17 and IL-17-related cytokine responses upon F4^+^ ETEC infection both in intestinal tissues and PBMCs. In a next effort we wanted to determine if these observations could be reproduced by oral immunization of piglets with F4 fimbriae, as these are potent oral immunogens. We only focused on the PBMCs since similar systemic and mucosal responses were found after F4^+^ ETEC infection. Upon immunization, the F4-specific IgG and IgA serum antibody titers were significantly increased as compared to the control group (Additional file 3), indicating a successful immunization. Similar to F4^+^ ETEC infection, oral immunization with F4 fimbriae significantly increased the mRNA expression of Th17-related genes, including IL-17F, IL-21, IL-22, IL-23p19 and RORγt, although with different kinetics (Figure 4). Compared to control pigs, significant changes were only observed at D4 for IL-17F and IL-23p19 and at D9 for IL-22, whereas IL-21 and RORγt mRNA expression levels peaked at D4. Unexpectedly, IL-17A mRNA expression was undetectable in porcine PBMCs upon oral immunization with F4 fimbriae. In contrast to the PBMCs of F4^+^ ETEC infected pigs, oral immunization with F4 fimbriae upregulated IL-17B mRNA expression at D4. In agreement with F4^+^ ETEC infection, only IFN-γ, but not IL-12 and T-bet, mRNA expression was elevated in immunized pigs. Likewise, GATA-3 levels increased in a time-dependent manner to reach significance at D9. Unexpectedly, we also failed to detect IL-13 mRNA expression in PBMCs upon F4 fimbriae immunization. Regarding the Tregs-related genes, only Foxp3 mRNA expression was enhanced. Similar to the infection trial, we also found a significant increase of AID mRNA expression, presumably indicating the presence of F4-specific circulating B-cells undergoing class switching.

### F4 fimbriae induced a Th17-signature cytokine expression in naive PBMCs

**Figure 5:**
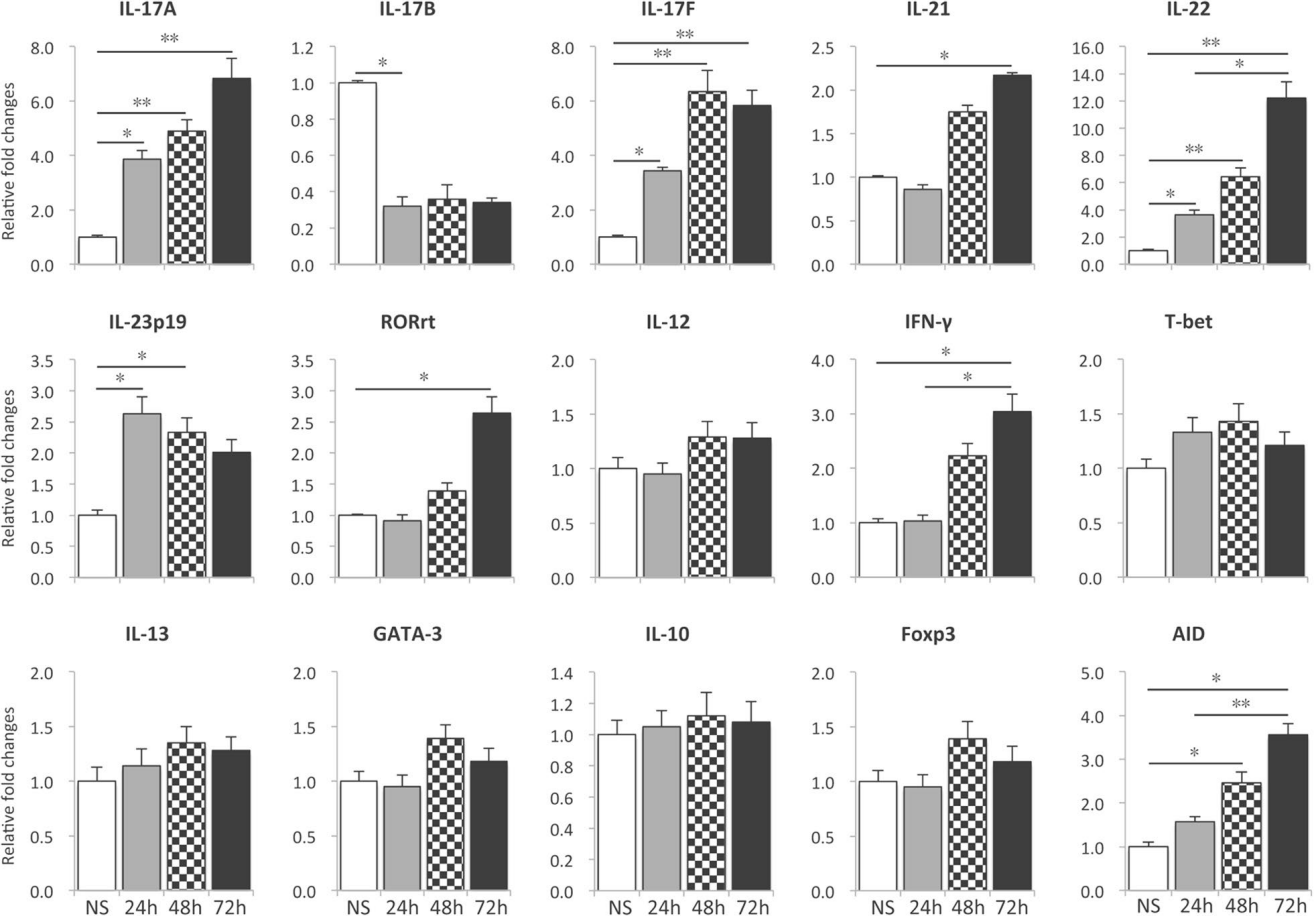
**F4 fimbriae elicit an IL-17 dominated cytokine response in naïve PBMCs**. PBMCs were isolated from naïve piglets and were stimulated with F4 fimbriae (5 μg/mL) for 24, 48 and 72 h. The mRNA expression profile was analyzed by qPCR. mRNA expression levels were normalized to the reference genes and then to the control group for each time point. Then, the data for every day was plotted relative to day 0. Data are presented as the mean ± SEM (*n* = 3). NS: non-stimulated. * *p* < 0.05, ** *p* < 0.01.

The above data indicate the potential of F4 fimbriae to induce Th17 responses. To further address this potential, PBMCs were isolated from naive animals and stimulated with F4 fimbriae. Upon stimulation for 24 h the mRNA expression levels of IL-17A, IL-17F and IL-22 were significantly increased as compared to unstimulated cells (Figure 5). This upregulated expression level lasted at least till 72 h post stimulation. In addition, F4 fimbriae significantly enhanced IL-23p19 mRNA expression, which peaked at 24 h post stimulation, while IL-21 and RORγt mRNA expression only displayed a significant change at 72 h (Figure 5). Intriguingly, F4 fimbriae stimulated PBMCs downregulated IL-17B mRNA expression. Regarding the Th1-related genes, we only observed a significant upregulation of IFN-γ transcripts, while IL-12 and T-bet mRNA levels were not affected. Furthermore, no significant difference in the mRNA expression of Foxp3, IL-10, GATA-3 and IL-13 was observed. Similar to the infection and immunization experiment, AID mRNA levels were significantly increased in F4 fimbriae stimulated PBMCs (Figure 5).

**Figure 6:**
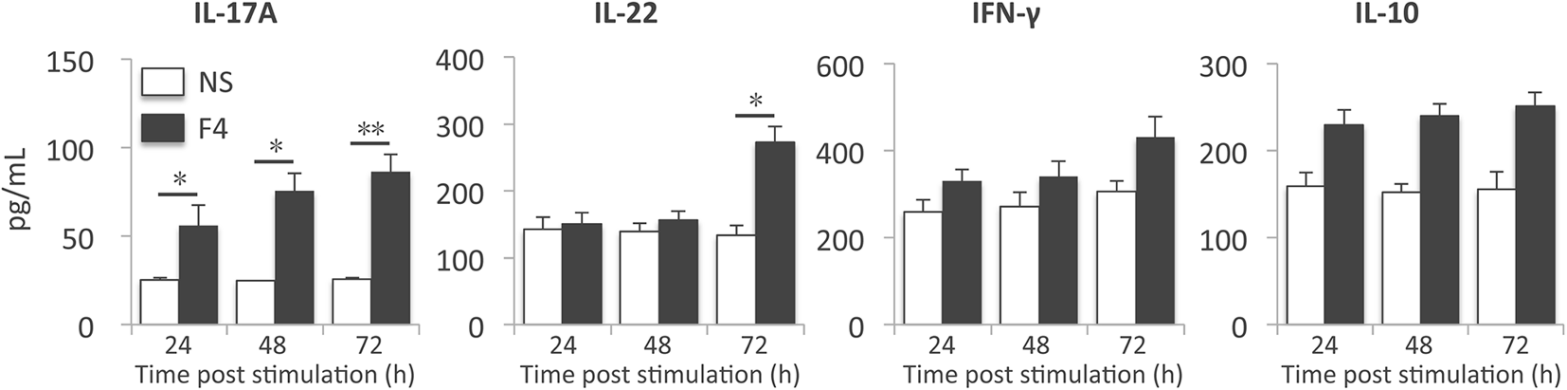
**Cytokine secretion by F4 fimbriae stimulated naïve PBMCs.** PBMCs were stimulated with endotoxin-free F4 fimbriae (5 μg/mL) or medium for 72 h. The protein level of IL-17A, IL-22, IFN-γ and IL-10 in the supernatant was determined by ELISA. Data are presented as the mean ± SEM (*n* = 3). NS: non-stimulated, * *p* < 0.05, ** *p* < 0.01.

To confirm that the increased transcript levels upon stimulation of PBMCs with F4 fimbriae resulted in higher protein levels, we measured the secretion of the corresponding cytokines using ELISA. As shown in Figure 6, stimulation with F4 fimbriae significantly increased IL-17A secretion by PBMCs as fast as 24 h post stimulation. Moreover, endotoxins in the fimbrial solution did not exert a significant effect on the IL-17A secretion (Additional file 4). F4 fimbriae also triggered the secretion of IL-22 by PBMCs, while no significant changes were observed for IL-10 and IFN-γ.

### F4 fimbriae boosted Th17 responses in an antigen recall assay

**Figure 7:**
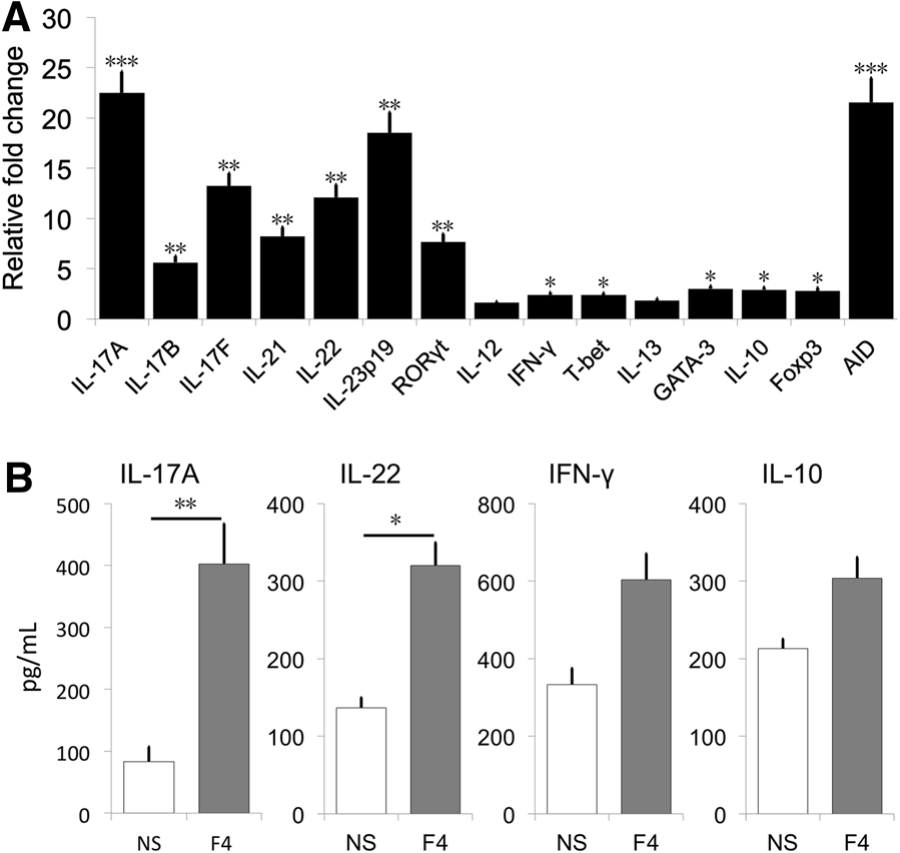
**Th17 signature dominates in an antigen recall assay.** PBMCs were isolated from F4^+^ ETEC infected animals and stimulated with F4 fimbriae (5 μg/mL) or medium for 48 h. **A** The mRNA expression profile in PBMCs was analyzed by qPCR. The mRNA expression level was normalized to the reference genes and then to control PBMCs. * indicates significant differences as compared to the control group. **B** The secretion of IL-17A, IL-22, IFN-γ and IL-10 by PBMCs was determined by ELISA. Data are presented as mean ± SEM (*n* = 3 per group). NS: non-stimulated, * *p* < 0.05, ** *p* < 0.01 and *** *p* < 0.001.

To confirm the presence of circulating F4-specific lymphocytes, PBMCs from F4^+^ ETEC infected pigs were restimulated with F4 fimbriae for 48 h and the mRNA expression levels of the above mentioned cytokines and transcription factors were assessed. As shown in Figure 7A, restimulation with the fimbriae resulted in the upregulation of all examined transcripts, except IL-13 and IL-12. The Th17-related genes IL-17A, IL-17B, IL-17F, RORγt, IL-21, IL-22 and IL-23p19 as well as AID were highly upregulated. In addition, Foxp3, IL-10, GATA-3, IFN-γ and T-bet mRNA expression levels were increased after F4 restimulation, although not to the same extent as the Th17-related cytokines. Furthermore, the upregulated IL-17A and IL-22 transcripts elicited by F4 fimbriae restimulation correlated with an augmented IL-17A and IL-22 cytokine secretion by the PBMCs, while the increase in IFN-γ and IL-10 production was not statistically significant (Figure 7B).

## Discussion

Th17 effector cells are important to eradicate mucosal pathogens including extracellular bacteria, fungi and even helminths [17, 39, 40]. These Th17 cells are characterized by the secretion of IL-17A, IL-17F, IL-21 and IL-22. The latter contribute to the protective function of Th17 cells by inducing the expression of defensins, mucins, tight junction proteins and lipopolysaccharide-binding proteins, which all reinforce the epithelial barrier [41]. Although some progress has been made to elucidate the cytokine response of intestinal epithelial cells to ETEC or its virulence factors [42–46], only few studies have evaluated the transcriptomic profile in PBMCs and intestinal tissues upon F4^+^ ETEC infection or oral immunization with F4 fimbriae prior to this report [47]. Recent work at our laboratory first hinted at the importance of IL-17A in innate immunity targeted to fend off F4^+^ ETEC infection as IL-17A mRNA levels were upregulated in the small intestine of piglets 4 h after F4^+^ ETEC colonization [33]. Zhu and colleagues on the other hand observed a downregulated expression of IL-17A mRNA in both jejunum and ileum 1 week after F4^+^ ETEC challenge, although this downregulation did not reach the significant level. In addition, no significant changes were observed in the mRNA expression levels of IFN-γ, IL-12p40, IL-4, IL-2, IL-10, Foxp3 and TGF-β, except for an upregulated IL-6 mRNA expression [34]. As F4^+^ ETEC colonize the gut very soon upon ingestion and clearance of this pathogen usually occurs at 7 days post infection [10], any changes in cytokine expression levels should have occurred earlier. Here, we evaluated the mRNA expression profile in PBMCs from day 0 to 4 upon F4^+^ ETEC infection and at day 4 in the intestinal tissues. F4^+^ ETEC infection triggered an increased expression of IL-17A, IL-17F, IL-21, IL-22, IL-23p19 and RORγt in the intestine and PBMC fraction, hinting at a potential role of Th17 cells to clear F4^+^ ETEC infections in piglets. Indeed, IL-17A, IL-17F, IL-21 and IL-22 are preferentially produced by Th17 cells, while the transcription factor RORγt and IL-23 play essential roles in the differentiation and expansion of Th17 cells, respectively [48, 49]. The involvement of Th17 cells is also evidenced by the occurrence of relatively large amounts of IL-17A producing T cells in the intestinal tissue of F4^+^ ETEC infected piglets. Interestingly, we also observed a strong upregulation of IL-17F mRNA expression. IL-17A and IL17F have similar biological actions as both cytokines mediate pro-inflammatory responses and play a role in the host defense against certain mucosal pathogens, such as *C.rodentium* [50–52]. Thus, we speculate that both IL-17A and IL-17F are required for the protection against F4^+^ ETEC infection in piglets. Further research should elucidate the contribution of each cytokine to protection against ETEC, especially as divergent roles for IL-17A and -F in immunity have been reported [53, 54]. Intriguingly, we observed a differential regulation of IL-17B mRNA expression in intestinal tissues and PBMCs upon F4^+^ ETEC infection, which may suggest IL-17B has a different function in mucosal and systemic immunity. Not much is known about IL-17B. This cytokine is expressed by monocytes and neutrophils and induces the secretion of pro-inflammatory cytokines [55–58]. Further research should identify the IL-17B-producing cells in pigs and elucidate their role in the host defense against mucosal pathogens.

In contrast to the increased level of Th17-related cytokines, F4^+^ ETEC infection did not affect the expression level of the Th1-related genes IL-12, IFN-γ and T-bet in both PBMCs and intestinal tissues, although a small increased IFN-γ expression was observed in the PBMC fraction on D3 and 4 upon infection. F4^+^ ETEC infection also resulted in an increased mRNA expression of IL-13, GATA-3, IL-10, and Foxp3, especially in the gastrointestinal tract. Upregulated Foxp3 and IL-10 mRNA expression probably indicates the induction of Tregs during the later stages of F4^+^ ETEC infection [59]. Since ETEC infection causes inflammation and intestinal damage in piglets, the induction of Tregs is probably required to limit these responses and to avoid immunopathology due to an overwhelming Th17 immunity [60, 61]. It is worth noting that F4^+^ ETEC infection triggered a significant increase in AID mRNA expression in PBMCs, Peyers patches and MLN. AID is a B cell specific enzyme required for the class switch recombination (CSR) in activated B cells [62]. In the F4^+^ ETEC infection model, F4-specific IgG and IgA antibody-secreting cells were observed in most tissues 4 days post infection [3, 10]. Hence, the increased AID mRNA level probably reflects ongoing class switching in B cells. Moreover, the strong induction of AID mRNA expression further supports the involvement of Th17 cells, since these cells also participate in B cell differentiation and subsequent SIgA production [13, 14, 63].

Previous studies in our lab indicated the strong oral immunogenicity of F4 fimbriae [3, 7, 9, 10]. To establish whether oral immunization with F4 fimbriae could elicit similar responses as an F4^+^ ETEC infection, we analyzed the cytokine mRNA expressions in PBMCs. Similar to infection, we observed a robust expression of the Th17-related genes RORγt, IL-23p19, IL-17F, IL-21 and IL-22. Likewise, Foxp3 and AID mRNA levels were also increased from day 4 onwards. These results indicate the capacity of F4 fimbriae to elicit Th17 responses and induce class switching in B cells upon oral administration. Unexpectedly, we failed to detect IL-17A mRNA expression. Upon ETEC infection, we observed a peak expression of IL-17A mRNA at 2 days post infection and the inability to detect IL-17A could be attributed to the later sampling point in the immunization experiment or indicate an important role for other molecules such as the enterotoxins in the IL-17A mRNA expression during infection [33]. The mRNA profile in the PBMCs upon oral immunization were F4 fimbriae specific, since PBMCs from naive piglets showed an upregulated mRNA expression of Th17-related genes and an enhanced secretion of IL-17A and IL-22 upon stimulation with these fimbriae. On top of that, these responses were further amplified in the PBMCs isolated from F4^+^ ETEC infected animals upon F4 fimbriae stimulation. In contrast, all the Th1- (except IFN-γ) and Th2-related genes did not show any significant change in naive PBMCs in the first 72 h, which corroborates a previous study [64]. In addition, Th1 effector cells and Tregs appear to be less important during F4^+^ ETEC infection, since no significant antigen specific IFN-γ and IL-10 recall responses were obtained by restimulation with F4 fimbriae.

In conclusion, F4^+^ ETEC infection and oral immunization with F4 fimbriae elicited robust expression of Th17-related genes and IL-17 producing T cells, indicating that Th17 effector cells participate in the protective immunity to ETEC infection in piglets and that these Th17 responses are in part induced by F4 fimbriae. Moreover, we also hinted at the potential participation of IL-17B and IL-17F in the clearance of F4^+^ ETEC infection. Altogether, our results could facilitate the design of ETEC vaccines.

## Additional files

10.1186/s13567-015-0264-2 **F4** ^**+**^ **ETEC number detected in the faeces after** **F4** ^**+**^ **ETEC infection.** Piglets were orally inoculated with either 10^10^ CFU F4^+^ ETEC (Pig 4 to 6) in 10 mL PBS or only PBS (Pig 1 to 3) as control at day 0 and 1. Faecal samples were collected from day 0 to 4 and inoculated onto blood agar plates and then the number of F4-specific hemolytic *E. coli* colonies was determined by dot blotting the colonies on nitrocellulose membranes and detecting F4 fimbriae with an F4ac-specific MAb.
10.1186/s13567-015-0264-2 **The distribution of mRNA in intestinal tissues of control pigs.** The mRNA expression in intestinal tissues of control pigs was analyzed by qPCR. The mRNA expression was normalized to the reference genes and then to jejunum without Peyer’s patches. Data are presented as the mean ± SEM (*n* = 3 per group). JJ = jejunum without Peyer’s patches, JP = jejunum with Peyer’s patches, IL = ileum without Peyer’s patches, IP = ileum with Peyer’s patches, MLN = mesenteric lymph nodes. * *p* < 0.05, ** *p* < 0.01.
10.1186/s13567-015-0264-2 **Oral immunization with F4 fimbriae induced F4-specific serum antibodies. **The piglets were immunized with 1 mg F4 fimbriae on day 0 (D0), D1 and D2. Blood was drawn on D0 before immunization and D9 after immunization. The F4-specific antibody (Ab) titer was tested in serum using ELISA. Data are presented as the mean ± SEM (*n* = 5 per group).
10.1186/s13567-015-0264-2 **Effect of endotoxins on IL-17A secretion. **PBMCs were stimulated with endotoxin-free F4 fimbriae (5 μg/mL), F4 fimbriae (5 μg/mL) in the presence or absence of polymyxin B (PMB, 25 μg/mL) or medium for 72 h. The IL-17A concentration in the cell-free supernatant was determined by ELISA. Data are presented as the mean ± SEM (*n* = 3).
