# Supplementary figures and images for: F4+ ETEC infection and oral immunization with F4 fimbriae elicits an IL-17-dominated immune response
Source: Vet Res. 2015 Oct 21;46:121. doi: 10.1186/s13567-015-0264-2 (PMC4618862; doi:10.1186/s13567-015-0264-2)

## Slide 1
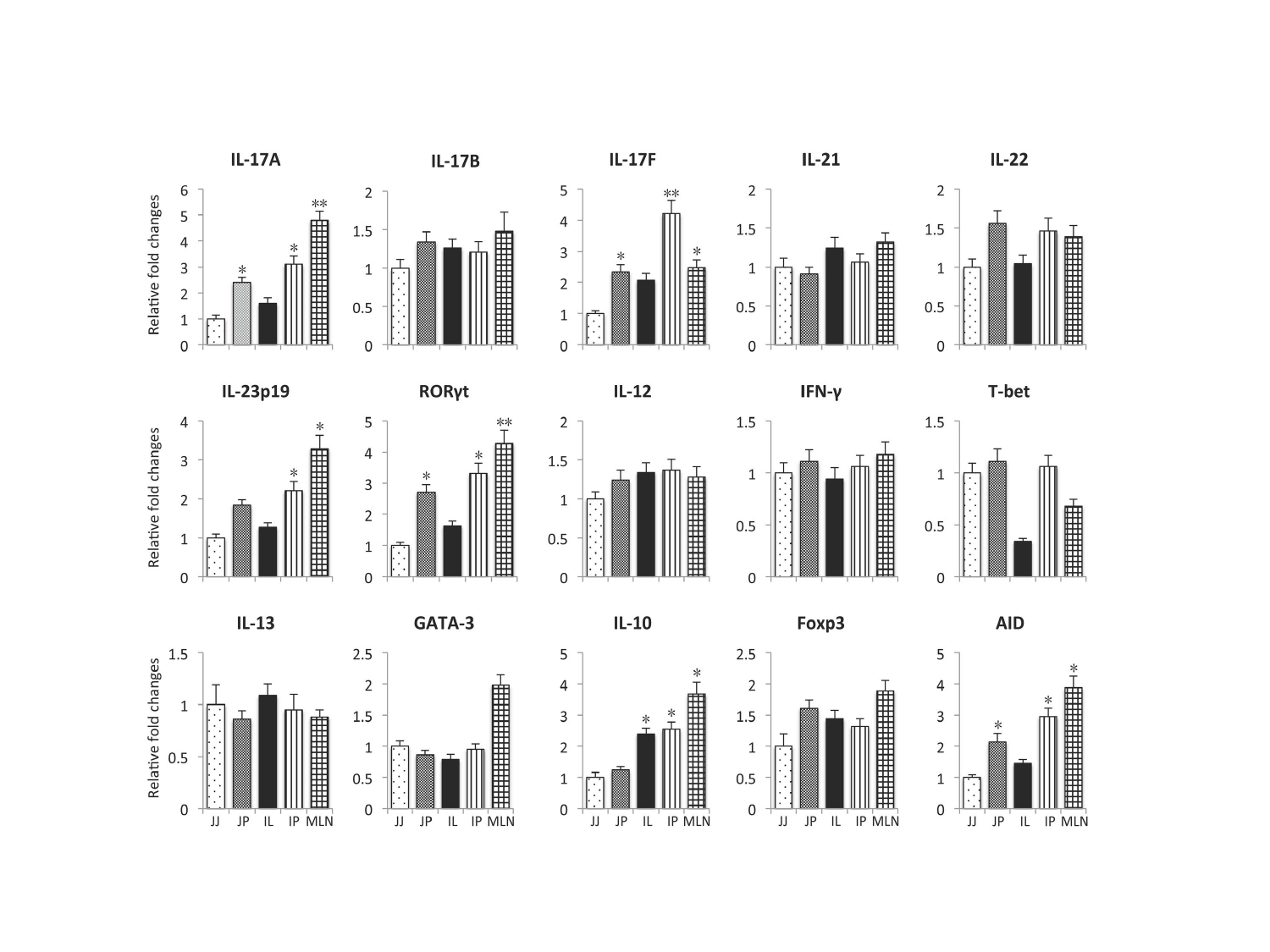

Supplement: Supplementary file 2 — 10.1186/s13567-015-0264-2 The distribution of mRNA in intestinal tissues of control pigs. The mRNA expression in intestinal tissues of control pigs was analyzed by qPCR. The mRNA expression was normalized to the reference genes and then to jejunum without Peyer’s patches. Data are presented as the mean ± SEM (n = 3 per group). JJ = jejunum without Peyer’s patches, JP = jejunum with Peyer’s patches, IL = ileum without Peyer’s patches, IP = ileum with Peyer’s patches, MLN = mesenteric lymph nodes. * p < 0.05, ** p < 0.01. [file 13567_2015_264_MOESM2_ESM.pptx]

## Slide 1
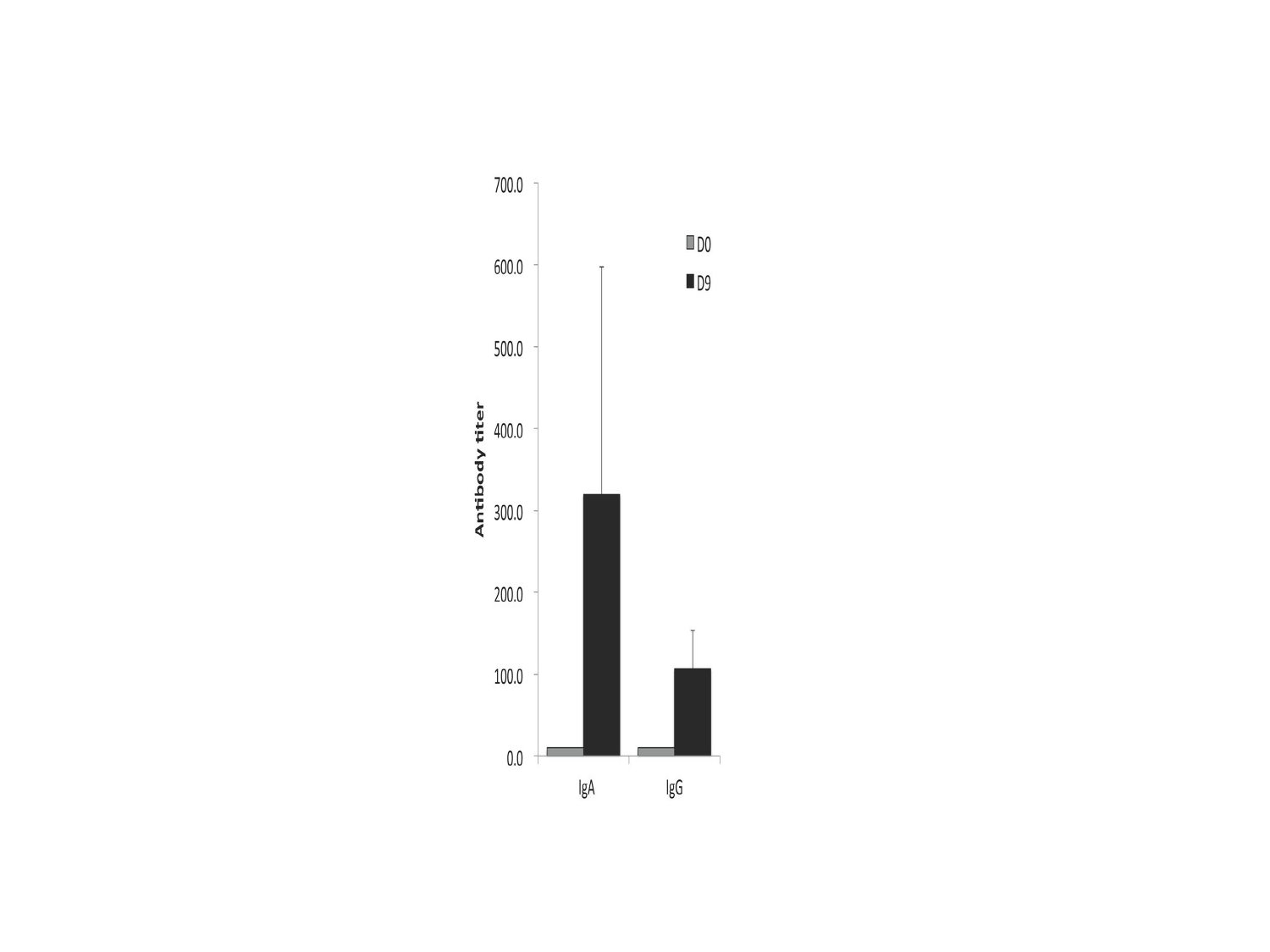

Supplement: Supplementary file 3 — 10.1186/s13567-015-0264-2 Oral immunization with F4 fimbriae induced F4-specific serum antibodies. The piglets were immunized with 1 mg F4 fimbriae on day 0 (D0), D1 and D2. Blood was drawn on D0 before immunization and D9 after immunization. The F4-specific antibody (Ab) titer was tested in serum using ELISA. Data are presented as the mean ± SEM (n = 5 per group). [file 13567_2015_264_MOESM3_ESM.pptx]

## Slide 1
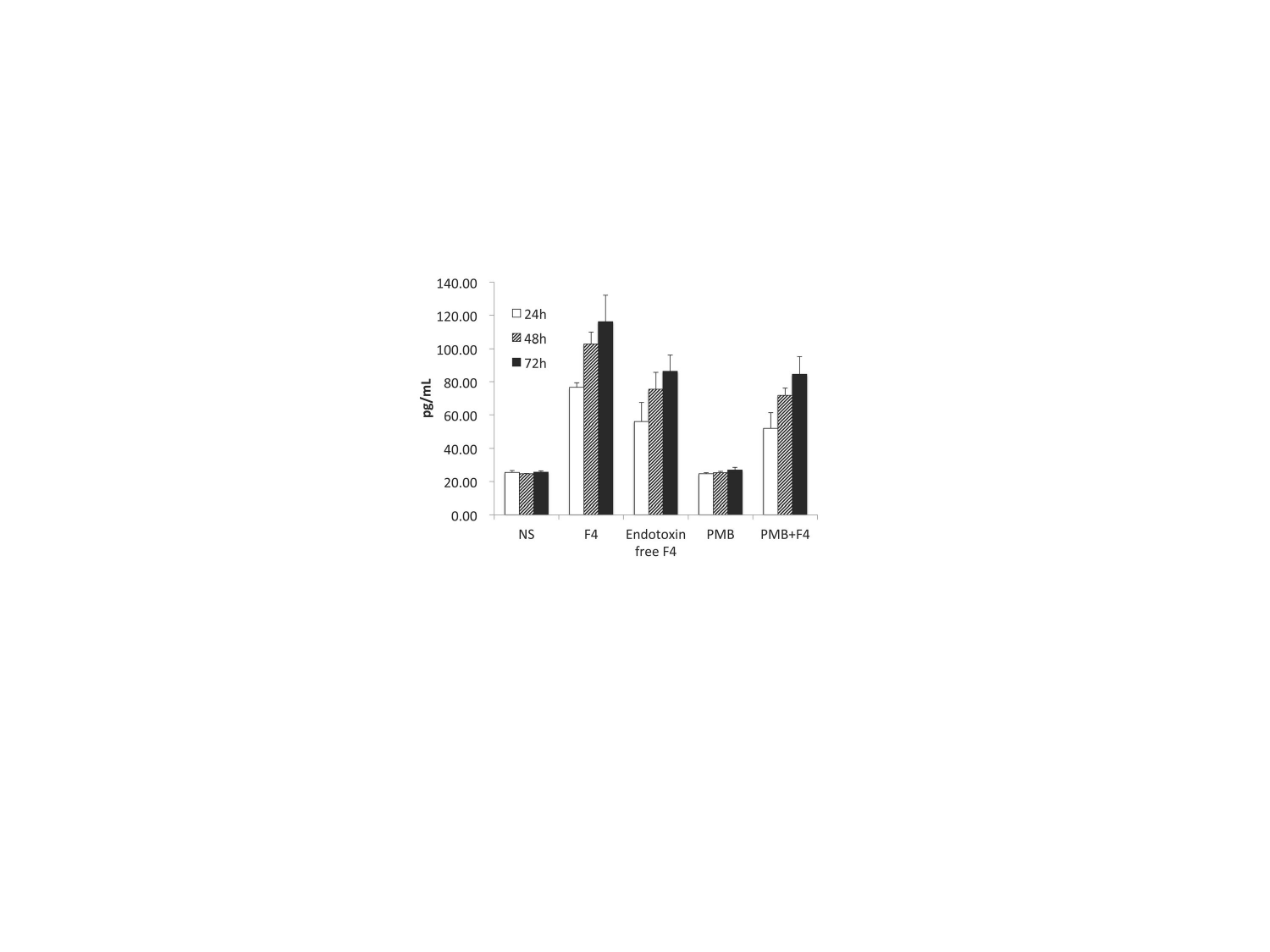

Supplement: Supplementary file 4 — 10.1186/s13567-015-0264-2 Effect of endotoxins on IL-17A secretion. PBMCs were stimulated with endotoxin-free F4 fimbriae (5 μg/mL), F4 fimbriae (5 μg/mL) in the presence or absence of polymyxin B (PMB, 25 μg/mL) or medium for 72 h. The IL-17A concentration in the cell-free supernatant was determined by ELISA. Data are presented as the mean ± SEM (n = 3). [file 13567_2015_264_MOESM4_ESM.pptx]
